# LVQ-SMOTE – Learning Vector Quantization based Synthetic Minority Over–sampling Technique for biomedical data

**DOI:** 10.1186/1756-0381-6-16

**Published:** 2013-10-02

**Authors:** Munehiro Nakamura, Yusuke Kajiwara, Atsushi Otsuka, Haruhiko Kimura

**Affiliations:** 1 Department of Natural Science and Engineering, Kanazawa University, Ishikawa 9200941, Japan; 2 Marine Faculty of Information Science and Technology, Ritsumeikan University, Shiga 5258577, Japan

**Keywords:** Biomedical data, Over-sampling, Learning Vector Quantization, Synthetic Minority Over-sampling Technique

## Abstract

**Background:**

Over-sampling methods based on Synthetic Minority Over-sampling Technique (SMOTE) have been proposed for classification problems of imbalanced biomedical data. However, the existing over-sampling methods achieve slightly better or sometimes worse result than the simplest SMOTE. In order to improve the effectiveness of SMOTE, this paper presents a novel over-sampling method using codebooks obtained by the learning vector quantization. In general, even when an existing SMOTE applied to a biomedical dataset, its empty feature space is still so huge that most classification algorithms would not perform well on estimating borderlines between classes. To tackle this problem, our over-sampling method generates synthetic samples which occupy more feature space than the other SMOTE algorithms. Briefly saying, our over-sampling method enables to generate useful synthetic samples by referring to actual samples taken from real-world datasets.

**Results:**

Experiments on eight real-world imbalanced datasets demonstrate that our proposed over-sampling method performs better than the simplest SMOTE on four of five standard classification algorithms. Moreover, it is seen that the performance of our method increases if the latest SMOTE called MWMOTE is used in our algorithm. Experiments on datasets for *β*-turn types prediction show some important patterns that have not been seen in previous analyses.

**Conclusions:**

The proposed over-sampling method generates useful synthetic samples for the classification of imbalanced biomedical data. Besides, the proposed over-sampling method is basically compatible with basic classification algorithms and the existing over-sampling methods.

## Background

With the arrival of big data society, the number of imbalanced biomedical data has increased, such as microRNA gene prediction [1] and detection of non-coding RNA [2]. Classification of imbalanced biomedical data has been one of the major issues in Bioinformatics. The common understanding of imbalanced data in the community is that the majority samples outnumber the minority samples [3]. The main problem of class imbalances is that most standard classification algorithms show poor classification performance because they assume or expect balanced class distributions.

Approaches to the class imbalance problem are broadly distinguished into two ways: one is “classification level” and another is “data level”. The classification level aims at adjusting the induction rules that describe the minority concepts which are often weaker than those of the majority concepts. One of the major approaches in the classification level is boosting [4]. The idea of boosting is to increases weights of misclassified samples and reduce the bias of class-imbalance learning. Another approach in the classification level is tree-based learning such as C4.5 [5] and Random Forest [6]. For example, the Random Forest classifier creates many of the minority concepts to avoid the biased learning.

The data level is the modification of an imbalanced dataset to obtain a balanced distribution. There are two major methods in the data level, namely over-sampling and under-sampling. The over-sampling method increases the samples in the minority class, while the under-sampling method decreases the samples in the majority class. Both of the methods aim at achieving a well-balanced class distribution. In general, the under-sampling method is used to reduce the learning time of a classification algorithm when the data size is larger enough to represent characteristics of the data, while the over-sampling method is used to increase the performance of a classification algorithm. Since approaches in the data level are independent from classification algorithms, approaches in the data level are more flexible than those in the classification level.

SMOTE (Synthetic Minority Over-sampling Technique) [7] is a powerful over-sampling method that has shown a great deal of success in class imbalanced problems. The SMOTE algorithm calculates a distance of the feature space between minority examples and creates synthetic data along the line between a minority example and its selected nearest neighbor. Han et al. developed a modified SMOTE called borderline-SMOTE [8]. The concept of their method is to generate synthetic samples near class boundaries. Their algorithms are specifically effective towards binary class problems with two features. However, since biomedical data such as gene expression data are often complex, they contain even thousands of features. Chen et al. presented an adaptive synthetic data generation called a RAMO technique [9]. They have shown in their experiments that the technique of an adapting boosting often increases the performance of the simplest SMOTE. Barua et al. developed a novel over-sampling method called MWMOTE [10], which generates synthetic samples in clusters of informative minority class samples. From their experiments, it is seen that MWMOTE outperforms RAMO and SMOTE on various benchmark datasets including biomedical data.

The existing over-sampling methods based on SMOTE achieve slightly better or sometimes worse result than the simplest SMOTE. One of the reasons is that even when an existing SMOTE is successfully applied to a biomedical dataset, its empty feature space is still so huge that it is difficult for classification algorithms to estimate proper borderlines between classes. As a solution to the problem, this paper presents a novel over-sampling method using codebooks obtained by LVQ (Learning Vector Quantization) [11]. The proposed method generates synthetic samples to occupy more feature space than the existing SMOTE algorithms.

## Methods

### Learning Vector Quantization

**Figure 1:**
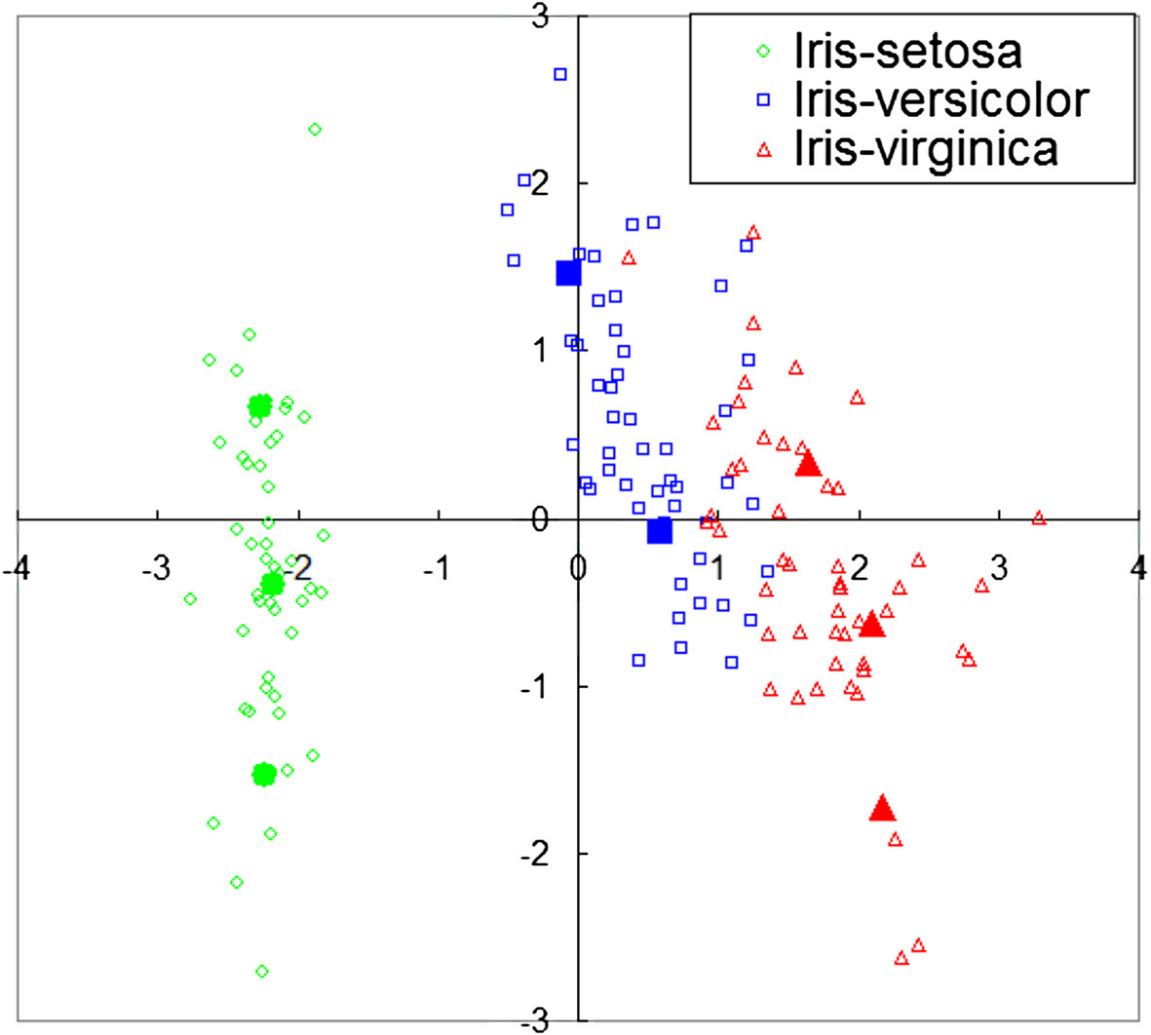
**Example of codebooks obtained by Learning Vector Quantization.** These codebooks are extracted from the samples in Iris dataset [12]. Each of the painted colored points represents the numerical value of a codebook.

LVQ is a supervised classification algorithm that has been widely used for various research purposes such as image decompression, clustering, and data visualization. LVQ is one of the neural networks modeled after the human’s visual cortex. Briefly saying, the algorithm of LVQ is a supervised version of *K*–means algorithm. As like *K*–means, the algorithm of LVQ determines a number of centroids called codebooks for each feature. Figure 1 shows an example of codebooks calculated by LVQ. The data in the figure are taken from Iris dataset (a benchmark dataset in UCI repository [12]), where the number of features is reduced from four to two by the principal component analysis. In the figure, each of the painted colored points represents the numerical value of a codebook. These codebooks are used to determine the class of an unknown sample according to the *k* nearest neighbor rule. Each codebook is randomly placed in the beginning and moves according to a rule based on the *K*–means algorithm.

There are various modified versions of LVQ developed by Kohonen, namely LVQ2.1, LVQ3, OLVQ3, Multiple-pass LVQ, Hierarchical LVQ [13]. Each of the algorithms is differ in how to determine the position of each codebook.

### The proposed over-sampling method

As described in the previous section, the codebooks for each feature in a target dataset are used to determine the class of an unknown sample. Hence, if the codebooks in the target dataset is similar to those in a reference dataset, it is expected that the samples in the reference dataset would provide the target dataset with informative data for its classification problem. From the idea, this paper presents a method of generating synthetic samples using real samples taken from reference datasets according to a similarity measure of codebooks.

**Figure 2:**
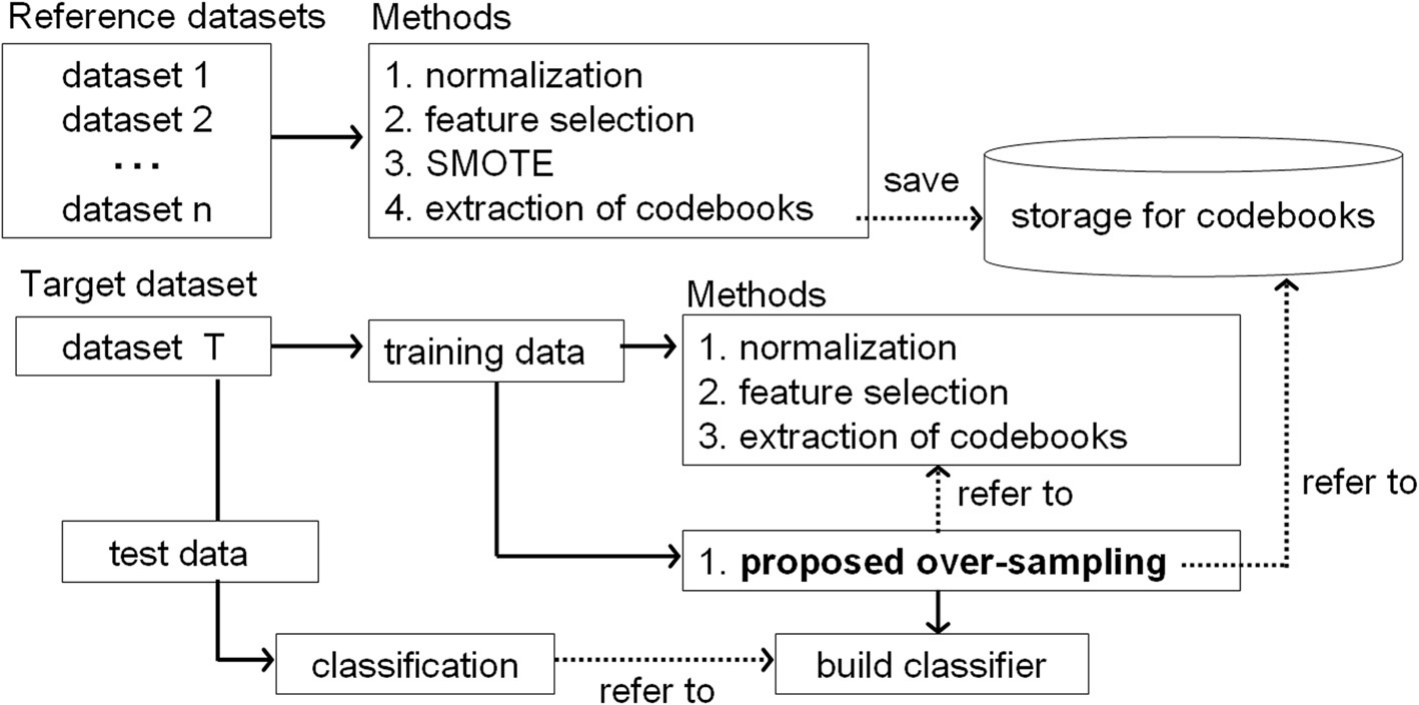
**Flow of the proposed over-sampling method.** The numbered methods are executed in ascending sequence.

**Figure 3:**
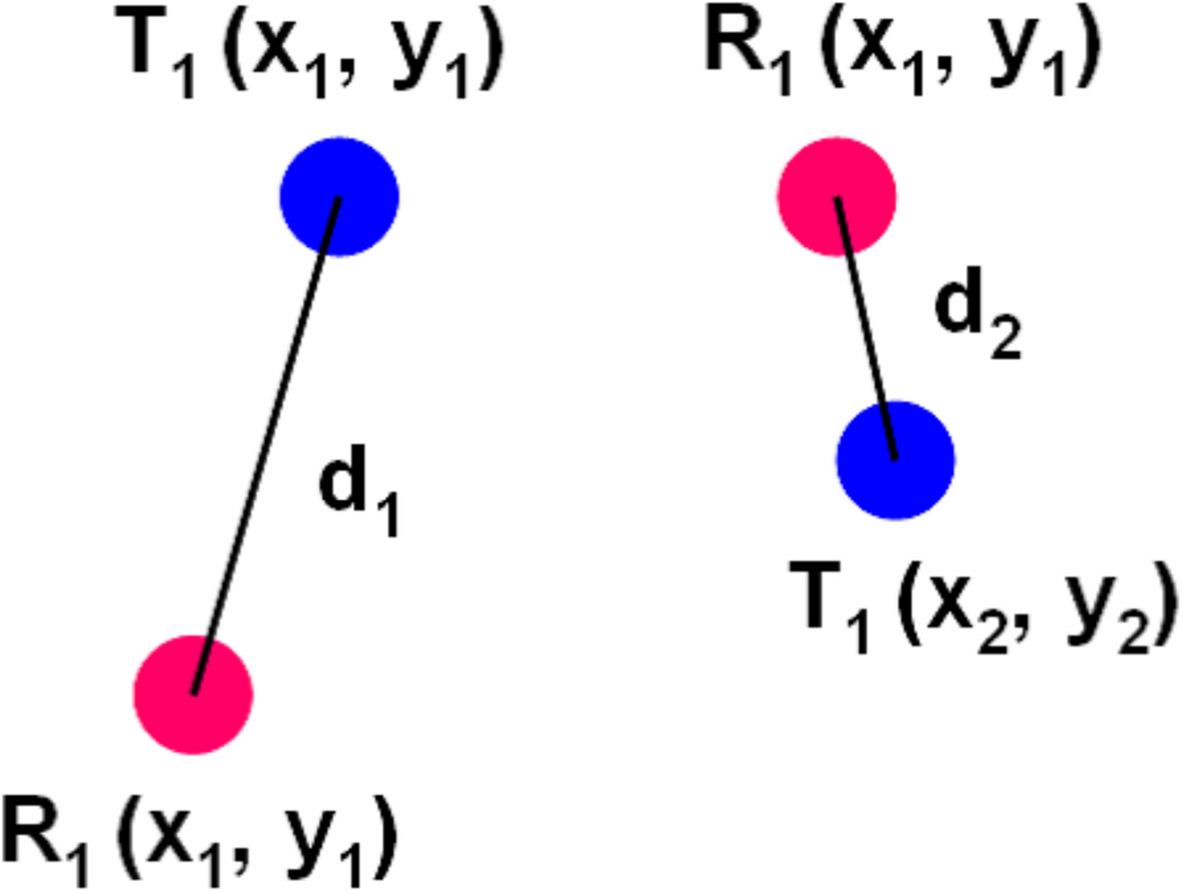
**Example of the distance measure.** The distance measurement is Euclidean distance.

Figure 2 shows a flow of the proposed method. As the figure shows, the proposed over-sampling method refers to a storage for codebooks extracted from reference datasets, and generates synthetic samples for a target dataset. First, we define the number of codebooks for each feature in the target dataset *T* as *n* and a set of two features in *T* as *T*_*i*_ (*i*=1,2,…,*nc*) where *nc* is the total number of the combinations of two features. Thus, each of *T*_*i*_ has *n* codebooks and two features. Next, regarding the numerical value of each codebook in *T*_1_ as *T*_1_(*x*_*j*_,*y*_*j*_) (*j*=1,2,…*n*), the sum of Euclidean distance between *T*_1_(*x*_*j*_,*y*_*j*_) and *R*_1_(*x*_*j*_,*y*_*j*_) of a reference dataset R is calculated. Figure 3 shows an example of Euclidean distances between *T*_1_(*x*_*j*_,*y*_*j*_) and *R*_1_(*x*_*j*_,*y*_*j*_). In this case, the sum of Euclidean distance between *T*_1_ and *R*_1_ is *d*_1_+*d*_2_. This procedure applies from *R*_1_ to all the set of two pairs in the storage. Then, *T*_1_ is linked to the set of two features which output the minimal sum of Euclidean distance.

**Figure 4:**
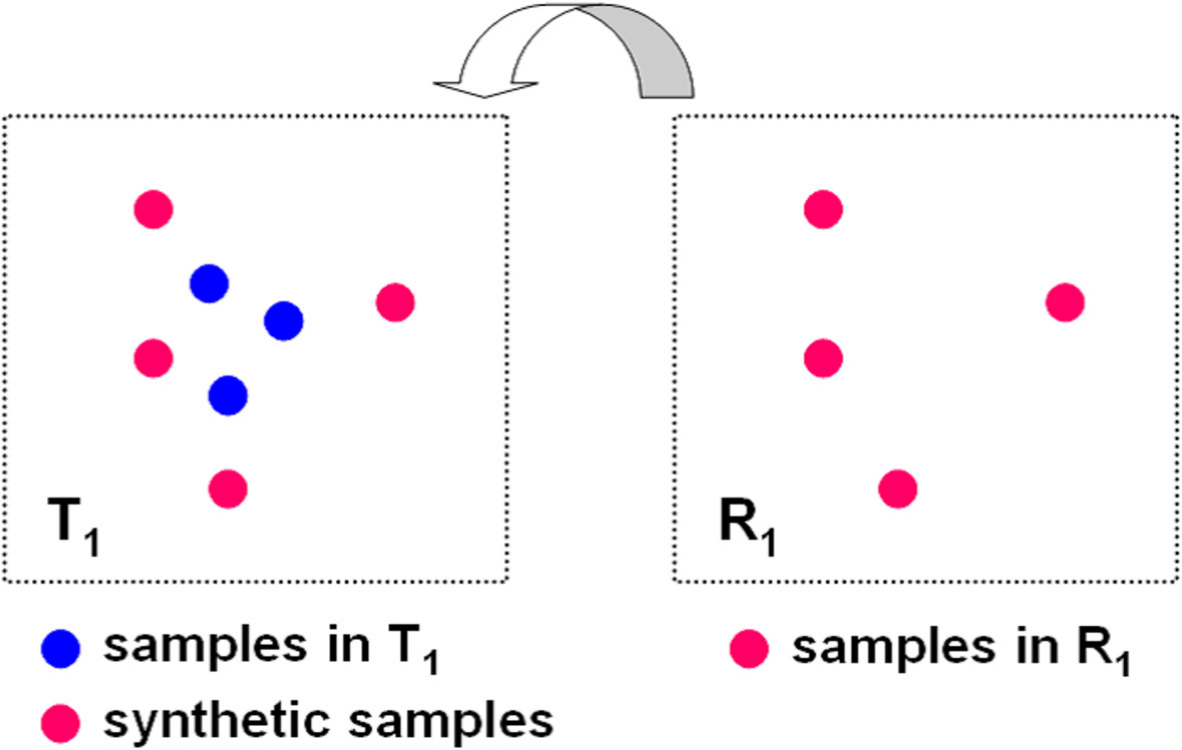
**Example of generated synthetic samples by our proposed method.** The four synthetic samples in *T*_1_ are the actual four samples taken from *R*_1_, where *T* is a target dataset and *R* is a reference dataset.

Here, we consider the case that *T*_1_ is linked to *R*_1_. Figure 4 shows an example of synthetic samples generated by our proposed method. As the figure shows, the samples in *R*_1_ is added to *T*_1_. If the dataset *T* has more than 3 features, the proposed method determines the numerical values for each of the other features by the following algorithm. 

•Find the nearest sample for each of the generated synthetic samples according to Maharanobis distance.

•The numerical values for each of the other features in the nearest sample are copied to those of the other features in the generated synthetic sample.

The procedures above are conducted for all the set of two features in the training dataset, namely from *T*_1_ to *T*_*nc*_. Finally, the SMOTE algorithm applies to *T* to obtain balanced class distribution.

## Results and discussion

### Datasets

In order to evaluate the classification performance of our method, we have prepared eight imbalanced benchmark datasets as shown in Table 1. In the table, the colon-cancer dataset provided by Alon et al. [14] is a gene expression dataset that aims at normal/abnormal classification of colon-cancer and consists of 62 colon tissue samples with 2000 features. The leukemia dataset [15] aims at the classification of 23 acute myeloid leukemia patients and 49 acute lymphocytic leukemia patients. The other six real-world datasets were obtained from UCI Machine Learning Repository [12]. As highly imbalanced problems, the satimage dataset and yeast dataset were converted into binary class problem: the class “damp grey soil” and the other classes in satimage, and the class “ME2” and the other classes in yeast. Except for satimage and ionosphere, the other datasets are biomedical data.

**Table 1:** Benchmark datasets used for our experiments

**Datasets**	**Features**	**Total samples**	**Imbalance ratio**
Breast-w	683	10	0.35 : 0.65
Blood	748	4	0.23 : 0.77
Colon-cancer	2000	62	0.35 : 0.65
Ionosphere	351	34	0.36 : 0.64
Leukemia	7129	72	0.34 : 0.66
Pima	768	8	0.35 : 0.65
Satimage	6435	36	0.097 : 0.903
Yeast	1484	8	0.034 : 0.966

Moreover, we performed *β*-turn types prediction on BT547 and BT823 dataset [16]. *β*-turns are classified into nine types based on the dihedral angles of the two center residues in the turn [17]. In this paper, we aim at improving prediction accuracy for DEBUT, which is one of the state-of-the-art methods for predicting *β*-turn types [18]. We obtained the datasets used for training and testing DEBT that are available online at http://comp.chem.nottingham.ac.uk/debt/.

### Parameter configuration for the proposed over-sampling method

As shown in Figure 1, the normalization and a feature selection method are executed in the proposed method. In our experiments, the normalization applied to change the range of feature values from 0 to 1 in the real number. And then, the principal component analysis, as the feature selection method, extracted 10 useful features according to the component scores in ascending order.

As the parameter of SMOTE techniques in the following section, five nearest neighbors were selected in their sample replacement. We selected Optimized Learning Vector Quantization 3 (OLVQ3) as a algorithm of LVQ, where the number of codebooks was configured with two.

### Classification algorithms

In order to demonstrate the versatility of our proposed method, we selected widely used basic classification algorithms, namely SVM (Support Vector Machine) [19], Logistic Tree [20], Neural Network [21], Naive Bayes [22], Random Forest [6], and OLVQ3. SVM was implemented using a package called LIBSVM [23], where all the parameters were set as default and Radial Basis Kernel was selected as the kernel. SVM is a powerful classification algorithm for two-class classification. The other algorithms were implemented using weka 3-7-9 package [24]. In the parameter configuration for these algorithms, since we aim at evaluating our over-sampling method, we focused on configuring them for gaining general performances, rather than optimizing them. After some preliminary runs, the number of trees in Random Forest was set as 200 and the number of codebooks in OLVQ3 was set as 600 to increase the performance of RF and OLVQ3, respectively, and all the other parameters were remained as default. In Weka 3-7-9, the default number of trees in RF is configured with 10, and we found 10 trees were insufficient to deal with several thousands of features in pre-experiments. Similarly, we increased the number of codebooks in OLVQ3 from the default value 20.

### Classification results on the eight imbalanced datasets

In order to estimate the classification performance for our proposed method and comparable methods, the 10-fold cross-validation was performed on each of the eight imbalanced datasets. For instance, we divided each dataset into two parts, namely 10% for testing and the rest 90% for training, while keeping the class distributions as possible as it is. We repeated the 10-fold cross-validation for 20 times in each trial, and calculated the average sensitivity, specificity, and G-mean, which are defined by the following terms, respectively.

(1) $$\begin{array}{l} \text{Sensitivity}=\frac{\text{TP}}{\text{TP}\;+\;\text{FP}} \end{array}$$

(2) $$\begin{array}{l} \text{Specificity}=\frac{\text{TN}}{\text{TN}\;+\;\text{FP}} \end{array}$$

(3) $$\begin{array}{l} G-\text{mean}=\frac{\text{Sensitivity}\;+\;\text{Specificity}}{2} \end{array}$$

were TP is the number of true positives (correctly identified as sick), FP is the number of false positives (incorrectly identified as sick), and TN is the number of true negatives (correctly identified as healthy).

First, the classification of the benchmark datasets was conducted to compare four cases: nothing (no-oversampling), AdaboostM1 [25], SMOTE, and the proposed over-sampling method (LVQ-SMOTE). Table 2 shows the average G-mean for each of the four cases. Except for the case of Neural Network, we can find that our proposed method outperforms both of AdaboostM1 and SMOTE. In this experiment, Logistic Tree output the highest G-mean among the standard classification algorithms. Here, Table 3 shows *Sensitivity*, *Specificity*, and G-mean calculated by Logistic Tree for each of the datasets. Although LVQ-SMOTE output worse *Sensitivity* than SMOTE in three of eight datasets, both of all the *Specificity* and G-mean in LVQ-SMOTE are superior to SMOTE. It is seen that our proposed method significantly improved the classification performance for colon cancer, ionosphere, and leukemia datasets.

**Table 2:** Average G-mean for three cases

	**G-mean**			
**Classification algorithm**	***Nothing*: base line**	**AdaboostM1**	**SMOTE**	**LVQ-SMOTE**
NaiveBayes	76.25%	77.34%	78.54%	78.94%
Logistic Tree	72.88%	74.21%	81.21%	83.64%
Neural Network	75.24%	79.62%	80.44%	80.24%
SVM	72.65%	73.31%	80.92%	83.22%
RandomForest	75.34%	78.96%	79.47%	80.68%
OLVQ3	75.76%	74.35%	80.88%	82.55%

*Nothing* represents that all the datasets were remained as the class imbalanced problem. In the case of SMOTE and LVQ-SMOTE, the minority samples were increased up to the number of the majority samples.

**Table 3:** Sensitivity, Specificity, and G-mean for each of the datasets

	***Sensitivity***		***Specificity***		**G-mean**	
**Datasets**	**SMOTE**	**LVQ-SMOTE**	**SMOTE**	**LVQ-SMOTE**	**SMOTE**	**LVQ-SMOTE**
Breast-w	76.40%	74.16%	64.21%	67.89%	70.31%	71.03%
Blood	95.44%	95.00%	97.38%	99.04%	96.41%	97.02%
Colon-cancer	80.00%	85.00%	63.64%	72.73%	71.82%	78.86%
Ionosphere	80.16%	86.51%	91.56%	92.44%	85.86%	89.48%
Leukemia	95.65%	100.0%	95.92%	100.0%	95.79%	100.0%
Pima	72.76%	71.27%	77.60%	80.20%	75.18%	75.73%
Satimage	78.75%	75.76%	68.53%	75.67%	73.64%	75.71%
Yeast	74.51%	71.72%	86.81%	90.81%	80.66%	81.27%

This is the case of Logistic Tree which has shown the highest G-mean among the basic classification algorithms in Table 2.

Table 4 shows G-mean for LVQ-SMOTE in case one of the latest over-sampling methods called MWMOTE [10] is used instead of SMOTE in our algorithm, where the classification algorithm used in this experiment is Logistic Tree. As the table shows, the G-mean for satimage has been increased by 1.30% by the use of MWMOTE in our algorithm, and 5 of 8 G-means have been improved by the use of MWMOTE in our algorithm.

**Table 4:** G-mean for our proposed method (LVQ-SMOTE) in case MWMOTE instead of SMOTE is used in our algorithm

	***Nothing*: baseline**		**Our proposed method**	
**Datasets**	**SMOTE**	**MWSMOTE**	**SMOTE**	**MWSMOTE**
Breast-w	70.31%	70.59%	71.03%	70.69%
Blood	96.41%	96.50%	97.02%	96.40%
Colon-cancer	71.82%	71.08%	78.86%	79.09%
Ionosphere	85.86%	85.92%	89.48%	91.28%
Leukemia	95.79%	95.92%	100.0%	100.0%
Pima	75.18%	74.07%	75.73%	75.69%
Satimage	73.64%	73.92%	75.71%	77.01%
Yeast	80.66%	81.20%	81.27%	81.38%

The algorithm used in this experiment is Logistic Tree.

### *β*-turn types prediction

As a classification algorithm, we used the SVM with optimized parameters configured in DEBT [18]. We applied our proposed method to the eight benchmark datasets as the reference to generate synthetic samples for the learning data, and seven-fold cross-validation was performed on the BT547 and BT823 dataset, respectively. In order to confirm the effectiveness of our method, SMOTE was not applied to the learning data. Table 5 shows MCC (Matthews Correlation Coefficient), *Sensitivity*, and *Specificity* obtained in the experiment. MCC is defined as below.

(4) $$\text{MCC}=\frac{\text{TP}\times \text{TN}-\text{FP}\times \text{FN}}{\sqrt{\left(\text{TP}+\text{FN})\times (\text{TP}+\text{FP})\times (\text{TN}+\text{FP})\times (\text{TN}+\text{FN}\right)}}$$

**Table 5:** **Results of** ***β*-turns prediction on the BT547 and BT823 dataset**

		**DEBT [**[18]**]**			**DEBT + our method**		
**Dataset**	***β*-turn type**	**MCC**	**Sensitivity**	**Specificity**	**MCC**	**Sensitivity**	**Specificity**
BT547	I	0.38	71.6%	82.6%	0.40	73.7%	85.0%
	II	0.33	63.0%	90.8%	0.31	66.7%	86.1%
	IV	0.27	69.8%	73.3%	0.38	81.6%	75.2%
	VIII	0.14	47.8%	84.4%	0.26	60.3%	84.1%
	Non-turn	0.37	21.1%	99.7%	0.39	30.4%	97.6%
BT823	I	0.39	70.6%	84.2%	0.37	71.3%	82.5%
	II	0.33	62.7%	91.2%	0.30	61.4%	92.1%
	IV	0.27	68.3%	74.4%	0.35	78.4%	78.9%
	VIII	0.14	42.2%	87.2%	0.17	47.9%	86.3%
	Non-turn	0.38	23.6%	99.7%	0.40	27.5%	97.4%

And, Table 6 shows MCC scores of DEBT + our method, DEBT, and one of the latest method for *β*-turns prediction. From Table 5, the average MCC was improved by 0.05 in BT547 and 0.016 in BT823, and the average *Sensitivity* was improved by 7.88% in BT547 and 3.82% in BT823 by using our method. Meanwhile, the average *Specificity* was slightly decreased, 0.56% in BT547. In Table 6, although MCC of our method on type I and II were lower than that of X.Shi et al., MCC of our method on type IV and VIII were higher than that of X.Shi et al. The type IV and VIII are rare patterns in *β*-turns prediction, and it was difficult to predict these types in the existing methods [18]. Since our method can be used to expand the feature space in a rare case. we can say that our method generated useful synthetic samples for type IV and VIII.

**Table 6:** **Comparison of MCC scores between DEBT + our method, DEBT, and another** ***β*-turn type prediction method**

**Dataset**	**Prediction method**	**I**	**II**	**IV**	**VIII**
BT547	DEBT + our method	0.40	0.31	0.38	0.26
	DEBT	0.38	0.33	0.27	0.14
	X.Shi et al. [26]	0.53	0.55	0.31	0.04
BT823	DEBT + our method	0.37	0.30	0.35	0.17
	DEBT	0.38	0.33	0.27	0.14
	X.Shi et al. [26]	0.64	0.63	0.32	0.13

## Conclusions

This paper has presented a new over-sampling method using codebooks obtained by Learning Vector Quantization. In general, even when an existing SMOTE is applied to a biomedical dataset, it is still difficult to estimate proper borderlines between classes. In order to tackle this problem, we have proposed to generate synthetic samples using codebooks obtained by the learning vector quantization. The experimental results on eight real-world benchmark datasets have shown that the proposed over-sampling method generates useful synthetic samples for the classification of imbalanced biomedical data. It is expected that the proposed over-sampling method is basically compatible with basic classification algorithms and the existing over-sampling methods. In addition, experiments on datasets for *β*-turn types prediction show our proposed method has improved prediction of *β*-turns type IV and VIII.

In the future work, we plan to analyze benchmark datasets for extracting more effective codebooks. Moreover, we would like to improve the proposed algorithm regarding the generation of synthetic samples.

## Competing interests

The authors declare that they have no competing interests.

## Authors’ contributions

MN designed and developed the over-sampling method, curried out the experiments, analyzed the results and drafted the manuscript. YK participated in the development of the over-sampling method and reviewed the manuscript. AO prepared the datasets used in the experiments and reviewed the manuscript. HK supervised the experiments and edited the manuscript. All authors have read, and approved the manuscript.
